# Time for change: integrating cranial ultrasound into routine practice in sub-Saharan Africa

**DOI:** 10.1038/s41390-024-03794-0

**Published:** 2025-01-07

**Authors:** Lizelle Van Wyk, Gugulabatembunamahlubi Tenjiwe Jabulile Kali

**Affiliations:** Department of Paediatrics and Child Health, Stellenbosch University, Cape Town, South Africa

Cranial ultrasound (CUS) remains one of the most commonly performed investigations in the neonatal intensive care unit (NICU). It provides low-risk, non-invasive, real-time, serial bedside neuroimaging and neuro-hemodynamics without requiring patient transport or sedation.^1,2^ It allows for the detection of a variety of central nervous system abnormalities—intraventricular hemorrhage (IVH), periventricular leukomalacia (PVL), congenital abnormalities, infections, post-hemorrhagic ventricular dilatation and hypoxic ischemic encephalopathy (HIE).^1^ It allows for timeous decision-making support for clinicians as well as enabling clinicians to provide parents with relevant information.^2^ Despite these advantages, it remains technology dependent, requiring knowledge, training and expertise.^1^ Although CUS is the standard of care in high income countries (HICs), this is not the case in sub-Saharan Africa (SSA), where technology is expensive and often lacking, with little training or expertise available.^3^

In this issue of Pediatric Research, Loucaides et al.^4^ have published a meta-analysis determining the prevalence and diversity of intracranial pathologies, as diagnosed by CUS, in SSA. The authors report a wide spectrum of pathologies- predominantly IVH, congenital abnormalities and central nervous system infections, with few studies exploring HIE.

The current review reports an approximate two-fold higher incidence of any grade and high grade IVH, as compared to HIC. Although this is an alarming statistic, it is likely an underrepresentation due to the lack of routine CUS performed, as well as the likelihood of mortality of many very low birth weight or extreme preterm infants who would have qualified for screening. It also has to be borne in mind that in many SSA countries, infants <1000 g are not offered full interventional care and many deliveries take place outside health facilities. This substantial IVH burden represents a significant potential for long-term poor neurodevelopmental outcome in a geographical location that can ill afford such increased medical burdens on health systems, society and parents. The early identification of pathology by CUS, in the absence of MRI, would be beneficial to enable limited resources to be directed appropriately.

SSA also carries the world’s highest burden of HIE, with few countries in SSA able to provide adequate, currently recommended therapy, despite evidence of a reduction in neurological burden.^5^ Despite the low use of CUS in HIE reported by Loucaides et al., CUS has been shown to adequately identify infants at risk of poor neurodevelopmental outcome after HIE.^6^ CUS may, therefore, represent a powerful tool for diagnosis and prognostication in HIE, especially in areas where access to MRI is lacking.

Predominantly data from South Africa and Nigeria were available for analysis in the review by Loucaides et al., indicating that research, equipment and personnel may be lacking on the rest of the sub-continent. This is concerning as SSA represents 30% of global deliveries with little decline expected over the next 20–50 years.^7^ The sub-continent also accounts for 26% of global preterm deliveries, with 9 of the 11 countries with the highest burden of prematurity residing in SSA.^8^ In SSA, neonatal deaths contribute 46% to the under-5 mortality rate, with the main causes attributable to preterm birth complications, intra-partum related events (including asphyxia), and various infective events.^9^ The potential for CUS to enhance neonatal care in SSA is therefore significant, with the prospects of contributing to decreasing the under-5 mortality rate on the sub-continent.

A prominent finding by Loucaides et al. was the lack of equipment in SSA, with many studies not reporting the type of ultrasound probes used or the use of probes not conventionally used for CUS. Accurate detection of CUS abnormalities are machine and probe dependant,^10^ highlighting the need for appropriate equipment in SSA. Although ultrasound use in all lower middle income countries is increasing, much of it has been aimed at the adult population and novel uses.^11^ The lack of neonatal-specific equipment and application is highlighted in the current review, with a Nigerian study showing that only 14% of ultrasound equipment was dedicated for pediatric use. Access to ultrasound machines, probes and reliable power supply as well as cost of equipment and competition for use at the healthcare facility, remain prominent barriers for its use for neonates.^3^

Although variable, CUS screening protocols for IVH and its sequelae require frequent scans and are continued until term-corrected age in HICs.^12^ Loucaides et al. showed that most studies for infants <32 weeks were performed within the first 2 weeks of life. Although this would suggest a decreased likelihood of missing IVH, it could mean that there may be an increased risk of missing the sequelae of IVH, which may only present after 14 days of life.^10^ Literature, however, has suggested that two normal CUS performed at 3–5 days and repeated at 10–14 days after birth have a 91–94% likelihood of remaining normal.^13^ This scanning regimen, which remains within the realms of possibility for resource restricted hospitals in SSA, offers an opportunity to improve clinical care and provide valuable parental information. Also included in Loucaides et al.’s review were studies in non-preterm infants performed for a variety of intracranial infections, hydrocephalus and HIE. CUS screening protocols for this population are not well established,^14^ and would require further evaluation for implementation in resource restricted hospitals in SSA.

MRI is rarely available in SSA. CUS scores have been developed aiming to predict neurodevelopmental outcome. These scoring systems, incorporating a range of ultrasound parameters, have shown a sensitivity and specificity of 74% and 91%, respectively, for predicting cerebral palsy and abnormal neurodevelopmental outcome.^15^ This may therefore further support the use of CUS in SSA for neurodevelopmental prediction.

In many of the studies incorporated in the review by Loucaides et al., CUS were performed by international researchers, with little evidence that CUS expertise has become engrained in the SSA medical fraternity for day-to-day neonatal management.^16^ This task-shifting is required, not only for the improvement of neonatal care but also for medical personnel empowerment. However, image interpretation is dependent on experience and relies on expertise, that is currently still lacking on the sub-continent. Collaborative relationships with foreign institutions and ultrasound schools are required to ensure that training and expertise are improved and that ultrasound practices in SSA align with international best practices.

As shown by Loucaides et al., intracranial pathologies are common in SSA and may be more prevalent than in HICs. Early identification of intracranial pathology has the potential to change clinical management and improve the short- and long-term outcome of infants in SSA. CUS services, expertise and equipment are significantly lacking on the sub-continent. There is an urgent need for cost-effective ultrasound technology, training programs, expertise support and infrastructure development in SSA to improve not only neonatal outcomes, but also decrease the under-5 mortality.
